# Neonatal Hepatic Myeloid Progenitors Expand and Propagate Liver Injury in Mice

**DOI:** 10.3390/jcm12010337

**Published:** 2023-01-01

**Authors:** Anas Alkhani, Cathrine Korsholm, Claire S. Levy, Sarah Mohamedaly, Caroline C. Duwaerts, Eric M. Pietras, Amar Nijagal

**Affiliations:** 1 Department of Surgery, University of California, San Francisco, CA 94143, USA; 2 The Liver Center, University of California, San Francisco, CA 94143, USA; 3 Department of Comparative Pediatrics and Nutrition, University of Copenhagen, 1870 Frederiksberg C, Denmark; 4 Department of Medicine, University of California, San Francisco, CA 94143, USA; 5 Division of Hematology, University of Colorado Anschutz Medical Campus, Aurora, CO 80045, USA; 6 The Pediatric Liver Center, UCSF Benioff Childrens’ Hospital, San Francisco, CA 94143, USA; 7 Eli and Edythe Broad Center of Regeneration Medicine, University of California, San Francisco, CA 94143, USA

**Keywords:** biliary atresia, perinatal liver injury, hematopoietic stem and progenitor cells, myeloid progenitor cells

## Abstract

Background: Biliary atresia (BA) is a progressive pediatric inflammatory disease of the liver that leads to cirrhosis and necessitates liver transplantation. The rapid progression from liver injury to liver failure in children with BA suggests that factors specific to the perinatal hepatic environment are important for disease propagation. Hematopoietic stem and progenitor cells (HSPCs) reside in the fetal liver and are known to serve as central hubs of inflammation. We hypothesized that HSPCs are critical for the propagation of perinatal liver injury (PLI). Methods: Newborn BALB/c mice were injected with rhesus rotavirus (RRV) to induce PLI or with PBS as control. Livers were compared using histology and flow cytometry. To determine the effects of HSPCs on PLI, RRV-infected neonatal mice were administered anti-CD47 and anti-CD117 to deplete HSPCs. Results: PLI significantly increased the number of common myeloid progenitors and the number of CD34^+^ hematopoietic progenitors. Elimination of HSPCs through antibody-mediated myeloablation rescued animals from PLI and significantly increased survival (RRV+isotype control 36.4% vs. RRV+myeloablation 77.8%, Chi-test = 0.003). Conclusions: HSPCs expand as a result of RRV infection and propagate PLI. Targeting of HSPCs may be useful in preventing and treating neonatal inflammatory diseases of the liver such as BA.

## 1. Introduction

Biliary atresia (BA) is the leading cause of pediatric liver transplants worldwide [1]. Though its exact etiology is unknown, the progressive perinatal liver injury (PLI) observed in patients with BA is caused by dysregulated immune responses to liver injury [2,3]. The rapid progression from liver injury to fulminant liver failure in children with BA suggests that factors specific to the perinatal hepatic environment are important for disease propagation. Therefore, understanding the perinatal hepatic immune environment in which inflammatory diseases such as BA develop and progress is important to identify promising treatment strategies for this devastating disease.

Our recent studies in mice indicate that the adaptive immune response plays a limited role in the pathogenesis of PLI [4], whereas the innate immune response, specifically myeloid populations, are critical for determining disease outcome as the relative proportions of pro-inflammatory and pro-reparative myeloid cells control disease severity [4]. 

Hematopoietic stem and progenitor cells (HSPCs) give rise to immune populations and reside in the liver of late-gestation human fetuses before emigrating to the bone marrow (BM) [5,6]; in mice, this transition occurs during the first weeks of postnatal life [7]. HSPCs react to inflammatory signals via Toll-like receptors, cytokines, and growth factors [8], and act as central hubs of inflammation by coordinating immune responses [9]. For example, IL-1ß binds to HSPCs and induces transcriptomic changes that skew HSPC differentiation towards myelopoiesis [8]. Through their rapid expansion and replenishment of mature myeloid populations, HSPCs are fundamental in facilitating the transition from inflammation to the resolution of liver disease [4,9,10,11]. Both human and murine models of liver injury show that monocytes derived from HSPCs transition from pro-inflammatory cells immediately after injury into pro-reparative monocytes once the injurious agent is no longer present [4,10,11,12]. Our group has previously elaborated on this observation by demonstrating that the abundance of pro-reparative Ly6C^Lo^ non-classical monocytes renders animals resistant to PLI and that reducing the number of Ly6C^Lo^ non-classical monocytes restores susceptibility to liver injury [4]. In addition to the rapid expansion of HSPCs and their differentiation into myeloid cells during inflammation, dysregulation of HSPCs can contribute to a feed-forward loop that leads to the pathologic expansion of inflammatory myeloid populations, resulting in chronic inflammation and tissue injury [9]. Taken together, these findings support the role of HSPCs and their mature myeloid progeny in propagating PLI. 

In this study, we hypothesized that HSPCs propagate PLI in neonatal mice. To test this hypothesis, we used an infectious model of PLI to examine the role of HSPCs in neonatal liver injury. Using this model, we compared HSPCs and mature myeloid populations from the liver and BM in the setting of homeostasis and PLI. Our results demonstrate that HSPCs expand during PLI and that depletion of HSPCs prevents liver injury. These findings support our hypothesis that HSPCs play an important role in propagating PLI.

## 2. Materials and Methods

### 2.1. Mice

BALB/c mice were obtained from the National Cancer Institute (Wilmington, MA, USA), and received humane care according to the Guide for Care and Use of Laboratory Animals. Mouse experiments were approved by the University of California, San Francisco Institutional Animal Care and Use Committee, and all mice were euthanized according to humane end points.

### 2.2. Creation of Single-Cell Suspensions

P3 and P14 livers were isolated and mechanically dissociated in phosphate-buffered saline (PBS). Juvenile (P14) livers underwent additional enzymatic digestion using 2.5 mg/mL liberase (Roche Indianapolis, IN, USA, 05401119001) in 1 M CaCl2 HEPES buffer. BM was isolated from the tibia, fibula, hip, and lower spines of neonatal (P3) and juvenile (P14) animals, and mechanically dissociated in PBS. Both liver and BM single-cell suspensions were filtered through a 100 um strainer prior to further analysis.

### 2.3. Flow Cytometry

Single-cell suspensions from the liver and BM were divided into two fractions. One fraction was stained for surface markers on mature myeloid cells (Supplementary Table S1). To isolate HSPCs, the other fraction was depleted of lineage-positive (Lin^+^) cells using a Direct Lineage Cell Depletion Kit (Miltenyi Biotec, Cambridge, MA, USA, 130-110-470) and stained for the following HSPCs: long-term hematopoietic stem cells (HSC^LT^), common myeloid progenitors (CMP^+^ and CMP^−^), and terminal myeloid progenitors (TMPs: megakaryocytic-erythroid progenitors, MEP; monocytic-dendritic progenitors, MDP; granulocytic-monocytic progenitors, GMP; granulocytic progenitors, GP; monocytic and common monocytic progenitors, MP) using cell surface markers (Supplementary Table S2). Flow cytometry was performed on a LSR Fortessa X20 (BD Biosciences, San Jose, CA, USA) and data were analyzed in FlowJo (Ashland, OR, USA).

### 2.4. Colony-Forming Unit (CFU) Assays

Single-cell suspensions from P3 and P14 livers and BMs were cultured on Metho-Cult^TM^ media containing methylcellulose, recombinant mouse stem cell factor, IL-3, IL-6, and recombinant human EPO. 2 × 10^4^ cells were plated and incubated at 37 °C in 5% CO_2_ for 12 days. The proliferation and differentiation ability of HSPCs was assessed by a blinded observer who categorized CFU into granulocyte, macrophage (GM), granulocyte, erythrocyte, macrophage, megakaryocyte (GEMM), and macrophage (M) colonies.

### 2.5. Postnatal Model of Perinatal Liver Injury

Rotavirus (RRV) was grown and titered in *Cercopithecus aethiops* kidney epithelial (MA104) cells. PLI was induced by intraperitoneal injections (i.p.) of 1.5 × 10^6^ focus forming units (ffU) RRV within 24 h of birth (P0). Controls were injected i.p. with PBS.

### 2.6. Histologic Analysis

Liver tissue was analyzed using immunohistochemistry (IHC) for CD34^+^ cells or hematoxylin and eosin (H&E). H&E slides were examined for signs of inflammatory infiltrate and tissue injury (e.g., necrosis). IHC slides were imaged at 40× magnification and CD34^+^ cells with large nuclei and little cytoplasm were counted as HSPCs by a blinded observer using QuPath [13]. Since CD34 is also present in vascular endothelial cells, all elongated cells with the morphologic appearance of endothelial cells were excluded [14]. The mean number of CD34^+^ cells/cm^2^ was calculated between all stained liver sections using QuPath [13].

### 2.7. Antibody-Mediated Myeloablation in Neonatal Mice

Myeloablation was induced by i.p. injections (20 µL) of anti-CD117 and anti-CD47. Anti-CD117 (0.20 µg/µL) was given only on day 0. Anti-CD47 was administered on day 0 (0.15 µg/µL), day 1 (0.20 µg/µL), day 2 (0.25 µg/µL), day 3 (0.30 µg/µL), and day 4 (0.35 µg/µL) post-RRV injection. Isotype controls were injected i.p. with isotype IgG2b (similar regimen as anti-CD117) and isotype IgG2a (similar regimen as anti-CD47). Escalating amounts of anti-CD47 and IgG2a were given to account for the natural increase in pup weight that occurs after birth. All antibodies for this experiment were purchased from BioXCell, West Lebanon, NH, USA.

### 2.8. Data Analysis

All graphs and statistics were generated using GraphPad Prism 9.3.1 (San Diego, CA, USA). Individual proportions of HSPCs were calculated based on absolute cell counts (ACC) as either a percentage (%) of the total lineage-negative progenitor compartment (Lin^−ve^ cells), or as a fraction of total HSC^LT^ and downstream myeloid progenitors (CMPs and TMPs). Mature myeloid cell proportions were calculated as a percentage of CD45^+^ leukocytes based on ACC. *p*-values were calculated using unpaired, non-parametric tests (Mann–Whitney was used to compare the proportion and ACC of HSPCs) except for survival comparisons that were performed using chi-squared. A *p*-value of <0.05 was considered significant. Error bars represent mean ± standard deviation (SD). All authors had access to the study data and reviewed and approved the final manuscript.

## 3. Results

### 3.1. The Liver Is a Reservoir for Hematopoietic Progenitors in Neonatal Mice

**Figure 1:**
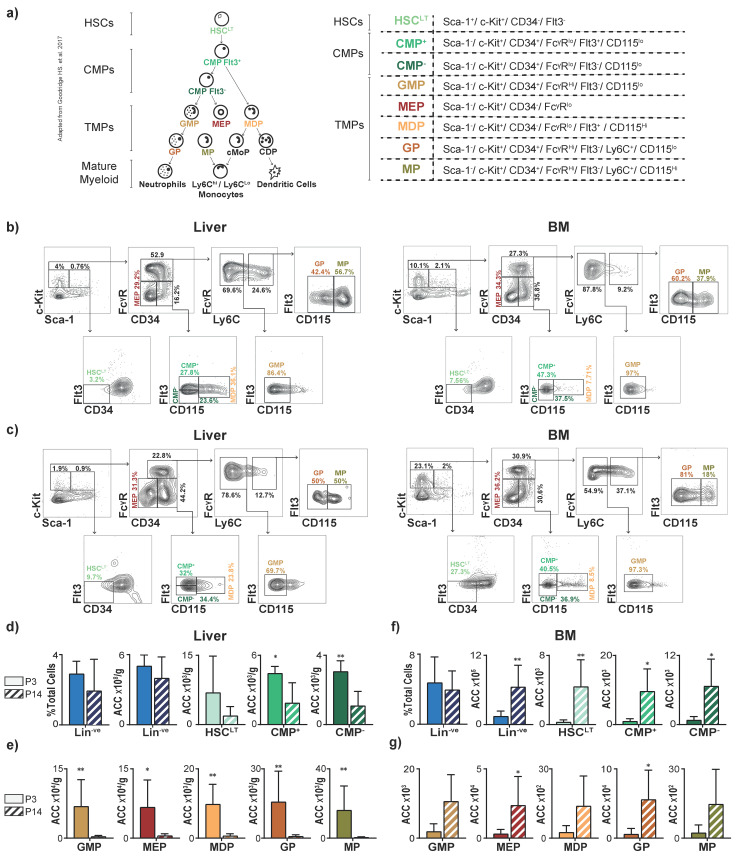
The liver is the main reservoir for myeloid progenitors in neonatal mice. (**a**) Schematic showing differentiation hierarchy and cell-surface markers of hematopoietic stem and progenitor cells (HSPCs): long-term hematopoietic stem cells (HSC^LT^), common myeloid progenitors (CMP^+^ and CMP^−^), and terminal myeloid progenitors (TMP: megakaryocytic-erythroid progenitors, MEP; monocytic-dendritic progenitors, MDP; granulocytic-monocytic progenitors, GMP; granulocytic progenitors, GP; monocytic and common monocytic progenitors, MP), and mature myeloid populations [15]. Plots demonstrating flow cytometric gating strategy of HSC^LT^s, CMPs, and TMPs in liver and bone marrow (BM) of PBS-injected mice on postnatal day 3 (P3) in (**b**) and postnatal day 14 (P14) in (**c**). Quantification of lineage negative (Lin^−^ve) fraction and absolute cell counts (ACC) of Lin^−ve,^ HSC^LT^, and CMP populations on P3 and P14 of life in (**d**) liver and (**f**) BM. Quantification of TMPs in P3 and P14 (**e**) liver and (**g**) BM. *n* = 6 for each group. *p*-value * < 0.05; ** < 0.01. Error bars represent mean ± SD.

To define the distribution of myeloid progenitors in neonatal animals under normal conditions, we quantified HSPCs (HSC^LT^s, CMPs, TMPs) and their mature progeny in the liver and BM of neonatal (P3) and juvenile (P14) mice. All HSPC populations were identified using cell-surface markers: HSC^LT^ were defined as Sca-1^+^. CMP and TMP populations were defined as Sca-1^−^. Individual CMP and TMP populations were distinguished based on the expression of CD34, FcγR, Flt3, Ly6C, and CD115 (Figure 1a–c) [15]. Since our previous work demonstrated a limited role for T- and B-lymphocytes in the pathogenesis of PLI, lymphoid progenitors were not quantified in this study [4].

We first quantified the Lin^−ve^ compartment in the liver and found that the percentage and number of Lin^−ve^ cells did not differ significantly between P3 and P14 livers (Figure 1d) and that the number of individual HSPC populations (CMPs and TMPs) were significantly lower at P14 compared to P3 (Figure 1d,e). 

In contrast to these findings in the liver, both the number of Lin^−ve^ cells and downstream progenitor populations (specifically HSC^LT^, CMP^+^, CMP^−^, MEP, and GP) in BM increased significantly between P3 and P14 (Figure 1f,g). In both the liver and BM, the mature myeloid populations mirrored the trends seen among progenitor populations as mature populations decreased in the liver and increased in the BM from P3 to P14; these differences, however, were not statistically significant (Supplementary Figure S1a,b). 

Collectively, these findings quantify the extent to which the murine liver retains hematopoietic progenitors during early neonatal life. These findings are also consistent with the known migration of hematopoietic progenitors from the liver to the BM during the first 2–3 weeks of postnatal life in mice [16]. 

### 3.2. The Juvenile Liver Retains Common Myeloid Progenitors and Myeloid Differentiation Capacity

**Figure 2:**
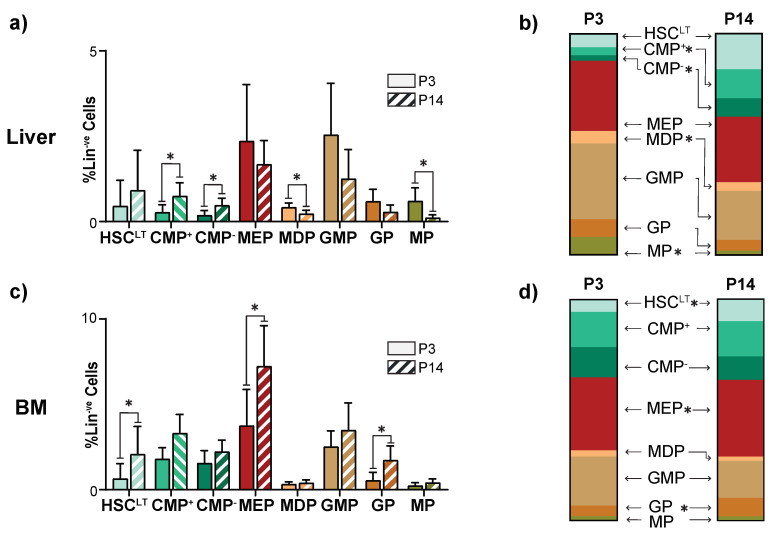
The juvenile mouse liver retains common myeloid progenitors. Percentage of hematopoietic stem cell (HSC^LT^), common myeloid progenitor (CMP), and terminal myeloid progenitor (TMP) populations among lineage negative (Lin^−ve^) cells in (**a**) liver and (**c**) bone marrow (BM) on postnatal day 3 (P3) and postnatal day 14 (P14). Relative proportions of HSC^LT^, CMP, and TMP among HSC^LT^ and their downstream myeloid progenitors in the (**b**) liver and (**d**) BM (P3, P14, *n* = 6). *p*-value * < 0.05. Error bars represent the mean ± SD.

We next asked whether the relative proportions of individual HSPC populations in the liver and BM changed from P3 to P14. In the liver, both the percentage of CMPs out of all Lin^−ve^ cells (Figure 2a) and CMPs as a fraction of total HSC^LT^s, CMPs, and TMPs (Figure 2b) significantly increased between P3 and P14. Meanwhile, all liver TMPs decreased, although this was only statistically significant for MDPs and MPs (Figure 2a,b). Unlike the liver, the BM exhibited a relative increase in the percentages of HSC^LT^s, CMPs, and TMPs out of total Lin^−ve^ cells between P3 and P14 (Figure 2c). This increase was only significant for HSC^LT^, MEPs, and GPs, and not for CMPs. The same trend was observed when we examined each population as a fraction of all HSC^LT^s, CMPs, and TMPs (Figure 2d). These results indicate that CMPs in the liver increase relative to other Lin^−ve^ cells, at a time when the main site of hematopoiesis is transitioning to the BM.

To test the differentiation potential of HSPCs from the liver and BM, we quantified colony-forming units of pro-myeloid colonies. The liver retained a similar pro-myeloid differentiation capacity at P14 compared to P3 as the number of granulocyte monocyte (GM) and granulocyte, erythrocyte, monocyte, megakaryocyte (GEMM) and megakaryocytes (M) colonies remain unchanged (Supplementary Figure S2a). In P14 BM, however, colonies from GM and GEMM increased, while the number of M colonies remained constant compared to P3 (Supplementary Figure S2b). 

The increase in CMPs in the livers of juvenile mice and the maintenance of myeloid differentiation capacity led us to question whether HSPCs residing in the liver play a role in perinatal liver injury.

### 3.3. Perinatal Liver Injury Leads to Expansion of CMPs in the Neonatal Liver

Based on our observation that the liver is a reservoir for HSPCs in neonatal mice and the known role of HSPC populations as central hubs of inflammation, we hypothesized that HSPC populations in the liver would expand during PLI. We have previously used rhesus rotavirus infection (RRV) in neonatal mice to study the role of immune populations during PLI [4]. Neonatal pups injected with RRV within the first 24 h of life develop progressive liver injury and a periportal inflammatory infiltrate that resembles the histological findings observed in human BA [17]. 

**Figure 3:**
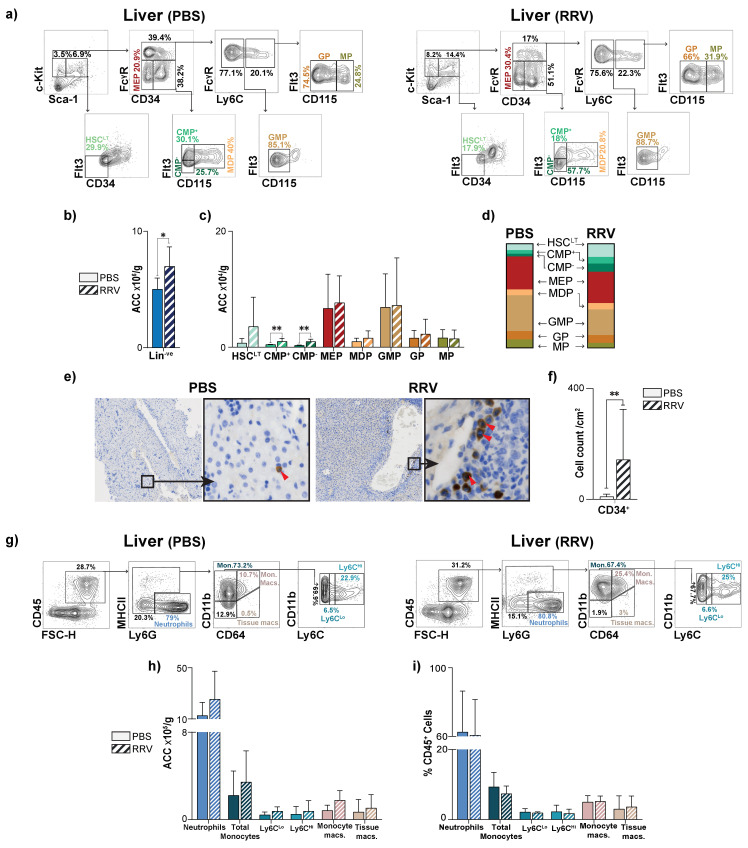
Perinatal liver injury results in the expansion of myeloid progenitors in the neonatal liver. (**a**) Representative flow plots demonstrating gating strategy of hematopoietic stem cells (HSC^LT^), common myeloid progenitors (CMP), and terminal myeloid progenitors (TMP) in liver of PBS- and RRV-injected 3-day-old (P3) mice. Quantification of absolute cell counts (ACC) of (**b**) lineage negative (Lin^−ve^) cells and (**c**) HSC^LT^s, CMPs, TMPs in P3 livers from PBS- (*n* = 6) and RRV-injected (*n* = 7) mice. (**d**) Relative fractions of HSC^LT^, CMP, and TMP populations in P3 livers from PBS- (*n* = 6) and RRV-injected (*n* = 7) mice. (**e**) Representative P3 livers in the setting of PBS and RRV with red arrows marking immunohistochemistry-stained CD34^+^ cells. (**f**) Quantification of CD34^+^ cells at P3 in PBS- (*n* = 6) and RRV-injected mice (*n* = 9). High-power images are at 40× magnification. (**g**) Representative flow plots demonstrating gating strategy of mature myeloid CD45^+^ populations in liver of PBS- and RRV-injected 3-day-old (P3) mice. (**h**) Quantification of ACC of mature myeloid populations in the liver of PBS- (*n* = 3) and RRV-injected (*n* = 5) mice at P3. (**i**) Quantification of %CD45^+^ leukocytes of mature myeloid populations in the liver of PBS- (*n* = 4) and RRV-injected (*n* = 8) mice at P3. *p*-value * < 0.05, ** <0.01. Error bars represent mean ± SD.

To evaluate the effects of PLI, we analyzed the livers of RRV-injected pups using flow cytometry and histology three days after injury (P3). All HSPCs were identified from flow plots as shown in Figure 3a. PLI significantly increased the number of Lin^−ve^ cells in the liver (Figure 3b), which was reflected in downstream HSC^LT^s and CMPs, though only CMPs reached statistical significance (Figure 3c). PLI had no effect on TMPs (Figure 3c). When we assessed each progenitor population as a fraction of total HSC^LT^s, CMPs, and TMPs, we found that PLI led to increases in liver HSC^LT^ and CMP fractions, though these differences did not reach statistical significance (Figure 3d). Using immunohistochemistry to localize CD34^+^ HSPCs in the P3 liver, we found that PLI significantly increased the number of CD34^+^ HSPCs/cm^2^ relative to controls and that these cells infiltrated all parts of the liver tissue with no identifiable pattern (Figure 3e,f). Notably, the expansion of CMPs did not lead to an increase in mature myeloid populations at P3 (Figure 3g–i).

**Figure 4:**
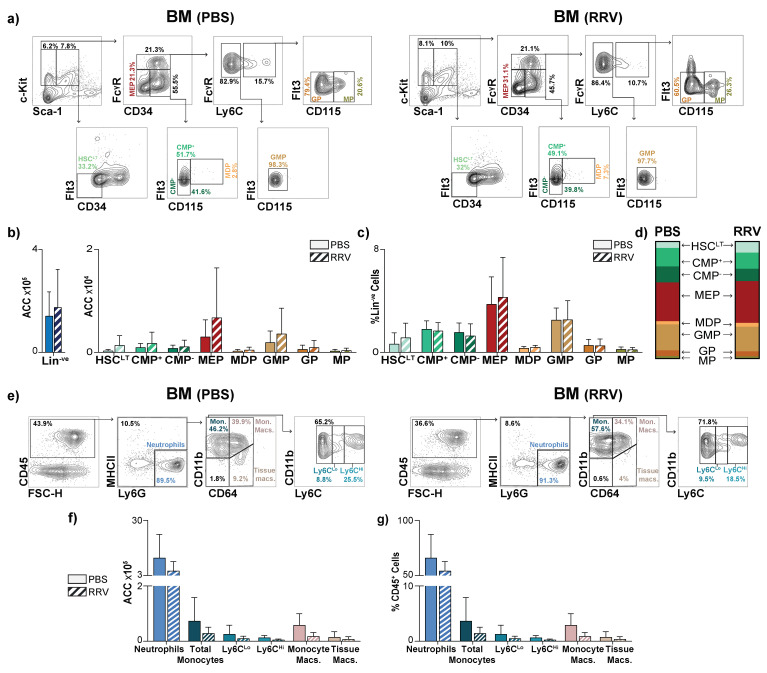
Perinatal liver injury causes no quantitative change to HSPCs in the BM of neonatal mice. (**a**) Representative flow plots demonstrating gating strategy of hematopoietic stem cells (HSC^LT^), common myeloid progenitors (CMP), and terminal myeloid progenitors (TMP) in BM of PBS- and RRV-injected 3-day-old (P3) mice. (**b**) Absolute cell counts (ACC) of lineage negative (Lin^−ve^) cells, hematopoietic stem cells (HSC^LT^), common myeloid progenitors (CMPs), and terminal myeloid progenitors (TMPs) of the BM, (**c**) percentage of total Lin^−ve^ cells, (**d**) fraction of HSC^LT^, CMPs, and TMPs. (**e**) Representative flow plots demonstrating gating strategy of mature myeloid CD45^+^ populations in BM of PBS- and RRV-injected 3-day-old (P3) mice. (**f**) ACC of mature myeloid populations (PBS *n* = 3, RRV *n* = 5) of the BM and (**g**) percentage of individual mature myeloid populations out of all CD45^+^ cells (PBS *n* = 3, RRV *n* = 5) at post-natal day 3 (P3). *n* = 6 for PBS and *n* = 6 for RRV unless otherwise stated. Error bars represent mean ± SD.

We questioned whether RRV infection affected HSPC populations residing in the BM of neonatal mice, and observed no significant changes in the absolute cell counts or percentages of HSPC populations in the BM after RRV infection (Figure 4a–c). Similar to the liver, we also did not identify significant changes to mature myeloid populations in the BM after RRV injection (Figure 4e–g). Taken together, RRV infection led to an expansion of CMPs specifically in the livers of neonatal mice.

### 3.4. Perinatal Liver Injury Leads to Contraction of HSPCs and Expansion of Mature Myeloid Populations in the Juvenile Liver

**Figure 5:**
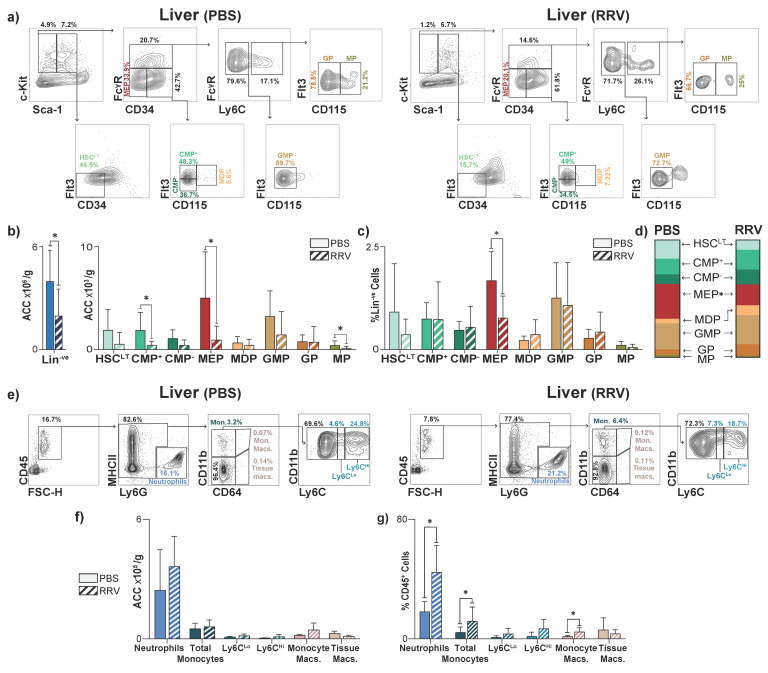
Perinatal liver injury leads to contraction of hematopoietic progenitors and expansion of mature myeloid cell proportions in the juvenile liver. (**a**) Representative flow plots demonstrating gating strategy of hematopoietic stem cells (HSC^LT^), common myeloid progenitors (CMP), and terminal myeloid progenitors (TMP) in liver of PBS- and RRV-injected 14-day-old (P14) mice (**b**) Absolute cell count (ACC) of lineage negative (Lin^−ve^) cells, hematopoietic stem cells (HSC^LT^), common myeloid progenitors (CMPs), and terminal myeloid progenitors (TMPs) of the liver, (**c**) percentage of total Lin^−ve^ cells, and (**d**) fraction of whole for HSC^LT^, CMPs, and TMPs. (**e**) Representative flow plots demonstrating gating strategy of mature myeloid CD45^+^ populations in liver of PBS- and RRV-injected 14-day-old (P14) mice. (**f**) ACC of mature myeloid populations (PBS *n* = 3, RRV *n* = 3) of the liver and (**g**) percentage of individual mature myeloid populations out of all CD45^+^ cells (PBS n = 5, RRV n = 9) at post-natal day 14 (P14). *n* = 6 for PBS and *n* = 8 for RRV unless otherwise stated. *p*-value * < 0.05. Error bars represent mean ± SD.

We next determined whether the expansion of CMPs seen in the neonatal liver after RRV infection was persistent or temporary. We quantified HSPC populations in juvenile mice 14 days after RRV infection (P14, Figure 5a) and we observed an overall reduction in Lin^−ve^ cells (Figure 5b). The number of HSPCs, including CMP^+^, MEPs, and MPs, decreased in RRV-infected P14 livers, compared to PBS-injected controls (Figure 5b). Despite the decrease in the number of these progenitor populations, MEP was the only progenitor population to decrease significantly as a percentage of Lin^−ve^ cells and as a fraction of total HSC^LT^s, CMPs, and TMPs (Figure 5c,d). While the number of all mature myeloid populations remained constant (Figure 5e,f), the percentage of neutrophils, monocytes, and monocyte-derived macrophages out of CD45^+^ leukocytes significantly increased in livers of RRV-infected juvenile mice, corresponding to the known peak of disease in RRV-infected animals (Figure 5e,g) [17]. These findings demonstrate that PLI causes a temporary expansion of CMPs in RRV-infected neonatal mice and that PLI results in a relative increase in mature myeloid populations 14 days after RRV infection.

### 3.5. Myeloablation Protects Mice from RRV-Mediated Perinatal Liver Injury

**Figure 6:**
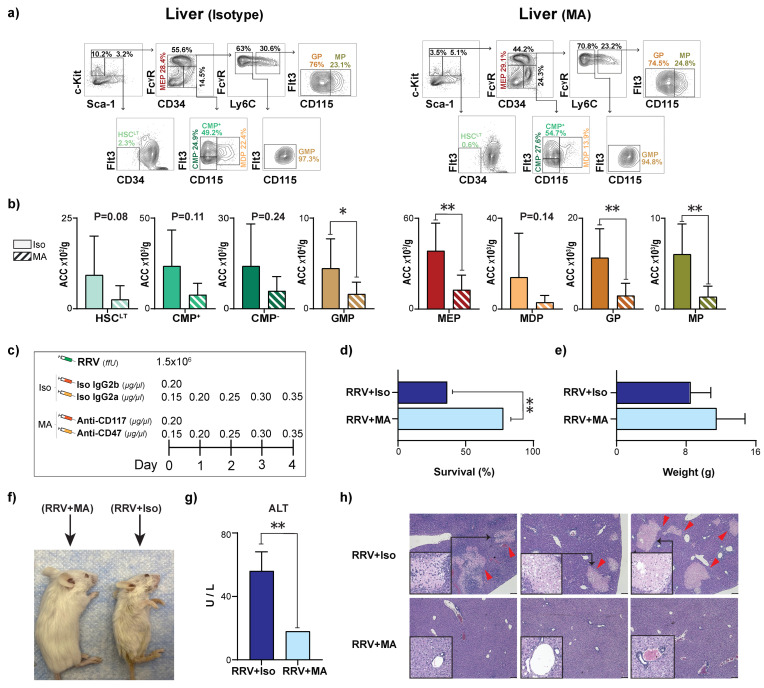
Antibody-mediated myeloablation depletes myeloid progenitor populations and protects mice from perinatal liver injury. (**a**) Representative flow plots demonstrating gating strategy of hematopoietic stem cells (HSC^LT^), common myeloid progenitors (CMP), and terminal myeloid progenitors (TMP) in liver of isotype (Iso)- and myeloablative (MA)-injected 3-day-old (P3) mice. (**b**) Quantification of absolute cell counts (ACC) of HSC^LT^s, CMPs, TMPs in P3 livers from Iso- (*n* = 6) and MA (*n* = 8) mice. (**c**) Dosage schedule illustrating rhesus rotavirus (RRV) injection, Iso, and MA. (**d**) Percent survival of RRV+MA (*n* = 27) vs. RRV+Iso (*n* = 22) injected controls at three weeks of life. (**e**) Pup weights of RRV+MA (*n* = 15) vs. RRV+Iso (*n* = 5) injected controls at three weeks of life. (**f**) Pictures illustrating phenotypic changes in mice after RRV+MA and RRV+Iso at three weeks of life. (**g**) Quantification of alanine transferase (ALT) in serum of RRV+MA (*n* = 3) treated mice vs. RRV+Iso (*n* = 3) injected controls at three weeks of life. (**h**) Histological H&E sections (5×) of livers from animals in both groups three weeks post-injection with either RRV + Iso or RRV + MA. Red arrows indicate necrotic foci. Black boxes indicate 20× magnified insets (necrotic foci are shown in RRV + Iso mice). *p*-value * < 0.05, ** < 0.01. Error bars represent mean ± SD.

The expansion of CMPs in the neonatal liver 3 days after RRV infection led us to question whether HSPCs play a role in propagating PLI. To test this, we evaluated the effect of depleting HSPCs on the progression of PLI, using synergistic, myeloablating anti-CD117 and anti-CD47 antibodies [18,19]. Myeloablation using anti-CD117 and anti-CD47 resulted in a reduction in all HSPC populations (Figure 6a,b). Though the reductions in HSC*^LT^* and CMPs did not reach statistical significance, four of the five downstream progenitors (GMP, MEP, GP, and MP) were significantly reduced after myeloablation (Figure 6a,b). We then tested the effects of myeloablation on RRV-infected mice. Neonatal pups were injected with RRV on day 0 to induce PLI. From day 0 to day 4, pups were also injected with anti-CD117 + anti-CD47 (MA) or IgG2b + Ig2a isotype controls (Iso) (Figure 6c). In the MA group, 78% of the pups survived RRV-mediated liver injury compared to only 36% in the isotype control group. (Figure 6d). MA pups also weighed more than isotype controls, but this difference was not statistically significant (Figure 6e). The improvement in survival and weight of the MA pups was corroborated by fewer moribund features, such as hair loss, dehydration, hunched appearance, and jaundice (Figure 6f). Finally, the extent of liver injury significantly decreased in the MA pups, as evidenced by lower levels of serum alanine transferase (Figure 6g), less periportal immune infiltrate, and fewer regions of hepatic necrosis (Figure 6h). 

These results indicate that the propagation of PLI after RRV infection is dependent on HSPCs.

## 4. Discussion

In this study, we defined the composition of HSPCs during homeostasis in neonatal and juvenile mouse livers, and we used an infectious mouse model of perinatal liver injury to define the changes that occur to HSPCs during liver injury. We found that (1) common myeloid progenitors reside in the livers of juvenile mice even after the main site of hematopoiesis has transitioned to the BM, (2) PLI leads to the expansion of common myeloid progenitors in neonatal mouse liver and causes an expansion of mature myeloid progenitors in juvenile mouse liver, and (3) targeted depletion of HSPCs using anti-CD117 and anti-CD47 prevents the development of RRV-induced PLI, as demonstrated by improved survival, increased jaundice clearance, and decreased liver injury.

Our results demonstrate the neonatal and juvenile mouse liver continues to act as a reservoir for common myeloid progenitors (CMPs) under homeostatic conditions. Furthermore, the differentiation capacity of HSPCs towards the myeloid lineage persists in the liver even after the main hematopoietic site has shifted to the BM. These findings corroborate the known role of the adult liver as a perpetual home for HSPCs [20]. The expansion of CMPs observed in P14 juvenile mouse livers also indicates that select HSPC populations may be important for the retention of myelopoiesis in the liver. Though the recruitment of myeloid cells (neutrophils, monocytes) during inflammation occurs from the bloodstream [12], our results also support the idea that remnant HSPCs in the liver may serve as central hubs of inflammation during PLI.

Our findings also support the idea that liver inflammation during perinatal life affects emigrating hepatic progenitor populations. In humans, the spatial and temporal overlap of liver development and hematopoiesis in the late-gestational fetus [21] may contribute to the devastating acute and chronic sequelae that affect liver- and immune function in progressive inflammatory diseases such as BA. In our study, perinatal liver injury led to an early increase in HSPCs and the depletion of these cells lessened clinical and histological signs of liver injury. Similar findings of HSPCs driving injury have previously been observed in the heart, where chronic inflammation directs HSPCs towards a pro-inflammatory phenotype that then enhances inflammation in a destructive feed-forward loop [9]. Our findings indicate that this detrimental feed-forward loop is similarly present in BA, where an injury to the fetal liver leads to dysregulation of HSPCs and propagation of tissue injury [22]. This theory is supported by the ‘layered hygiene hypothesis’ which suggests that fetal-derived HSPCs contribute to adult immune function and consequently, that impairment of fetal hematopoiesis can change the long-term trajectory of the immune system, potentially causing both autoimmunity and increasing disease susceptibility [23].

The current treatment of BA relies on early surgical treatment to restore bile flow after Kasai portoenterostomy, although most patients will continue to develop progressive liver injury requiring liver transplantation, highlighting the need for new and innovative treatments. Our results indicate that HSPCs propagate PLI in mice and suggest that HSPCs may also contribute to human BA. Intriguingly, the manipulation of HSPC populations has been found to influence disease outcome in human patients. In infants with BA who have undergone Kasai portoenterostomy, the effect of administering three consecutive days of granulocyte-colony-stimulating-factor (G-CSF) on liver inflammation was examined in a phase 1 trial demonstrating that peripheral neutrophils and HSPCs initially increased before decreasing to baseline levels after two weeks. Notably, G-CSF treatment was associated with reduced cholestasis one month after treatment but reverted to control levels after three months [24]. A phase 2 randomized controlled trial is currently underway to determine the efficacy of G-CSF in patients with BA (NCT04373941). Our findings combined with those from the phase I trial highlight that HSPC populations are dynamic during the course of an inflammatory insult and their functions change depending on the stage of disease and age of the patient. These findings also support the idea that manipulation of specific HSPC subsets may prove to be efficacious in resolving BA.

Our observed changes to HSPCs after RRV infection support the idea that early perinatal liver inflammatory insults have long-term consequences to immune function. Children with BA have an increased infection rate and decreased vaccine responses compared to healthy controls [25,26] and they are more likely than children with diseases other than BA who received liver transplants to reject donor livers [27]. In mice, a primary pathogenic stimulus has been found to cause changes in epigenetic and translational properties of HSPCs [28,29] resulting in a sustained myeloid lineage bias and an increased inflammatory response [8], which leads to a heightened response to similar secondary stimuli—a concept known as ‘trained immunity’ [28,29]. Maladaptive training of the myeloid compartment can result in increased susceptibility to other inflammatory conditions [8]. As myeloid cells account for the primary immune response during PLI [4], maladaptive trained immunity may similarly play a role in the long-term immune dysregulation observed in BA.

In our study, we eliminated HSCs and downstream hematopoietic progenitors using targeted antibodies directed against CD117 and CD47, thereby avoiding the devastating and non-specific tissue injury associated with traditional HSC-depleting strategies such as radiation and chemotherapy [18]. This approach does, however, have limitations that need to be addressed before considering its use in human patients. The intended removal of stem- and progenitor cells leads to a transient, secondary reduction in red blood cells [18]. Though previous studies have found anti-CD47 and anti-CD117 induced anemia to be mild and fully resolved within 2–3 weeks [30,31], further work will be needed to define the impact of neonatal anti-CD117 and anti-CD47 myeloablation on short and long-term anemia. Additional mild and temporary side effects of antibody-mediated myeloablation have also been reported, including hair color change and reduction in spermatogonia [18]. We expect that the short duration of myeloablation we used in neonatal mice would result in limited long-term toxicity; however, further studies would be needed to balance effective dosing and duration with side effects in human patients.

In conclusion, our study demonstrates that myeloid progenitors increase during PLI and that their depletion improves disease outcome. Future studies are necessary to investigate the specific effects of myeloablation on myeloid progenitor populations in neonates. Our study suggests that targeting hematopoietic progenitors may be useful in preventing and treating neonatal liver inflammatory diseases such as BA.

## Data Availability

The original contributions presented in the study are included in the article/supplementary material; further inquiries can be directed to the corresponding author.

## Supplementary Materials

The following supporting information can be downloaded at: https://www.mdpi.com/article/10.3390/jcm12010337/s1, Figure S1: Mature myeloid populations contract in the liver while expanding in the bone marrow of the juvenile mouse, Figure S2: HSPCs in the livers of juvenile mice maintain myeloid differentiation capacity, Table S1: Staining panel for mature myeloid cells, Table S2: Staining panel for hematopoietic stem- and progenitor cells (HSPCs): Long-term hematopoietic stem cells (HSC^LT^), common myeloid progenitors (CMPs), and terminal myeloid progenitors (TMPs).

## Abbreviations

ACC	Absolute Cell Count
BA	Biliary Atresia
BM	Bone Marrow
CFU	Colony Forming Units
CMP	Common Myeloid Progenitors
ffU	Focus Forming Units
G-CSF	Granulocyte-colony-stimulating-factor
GEMM	Granulocyte, Erythrocyte, Macrophage, Megakaryocyte
GM	Granulocyte, Macrophage
GMP	Granulocytic-monocytic Progenitors
GP	Granulocytic Progenitors
H&E	Hematoxylin Eosin
HSC^LT^	Long-term Hematopoietic Stem Cells
HSPC	Hematopoietic Stem and Progenitor cells
IHC	Immunohistochemistry
Iso	Isotype
Lin^−ve^	Lineage negative hematopoietic compartment
Lin^+^	Lineage positive hematopoietic compartment
Ly6C^Hi^	Ly6c^Hi^ classical monocytes
Ly6C^Lo^	Ly6C^Lo^ non-classical monocytes
M	Macrophage
MA	Myeloablation
MDP	Monocytic-dendritic Progenitors
MEP	Megakaryocytic-erythroid Progenitors
MP	Monocytic and common monocytic Progenitors
PLI	Perinatal Liver Injury
RRV	Rhesus Rotavirus
TMP	Terminal Myeloid Progenitors
