# Supplementary material for: Neonatal Hepatic Myeloid Progenitors Expand and Propagate Liver Injury in Mice
Source: J Clin Med. 2023 Jan 1;12(1):337. doi: 10.3390/jcm12010337 (PMC9821039; doi:10.3390/jcm12010337)
Supplement: Supplementary file 1 [file jcm-12-00337-s001.zip › jcm-2105144-supplementary.pdf]

Supplementary Material

Table S1. Staining panel for mature myeloid cells.

Antibody	Clone	Manufacturer	Catalog number
MHCII	M5/114.15.2	eBioscience, Waltham, MA	48-5321-82
Cd11b	M1/70	eBioscience, Waltham, MA	47-0112-82
Cd45	30-F11	eBioscience, Waltham, MA	56-0451-82
Ghost	*Not relevant*	Tonbo Biosciences, San Diego, CA	13-0870-T100
Ly6C	AL-21	BD Biosciences, Franklin Lakes, NJ	563011
Ly6G	1A8	BD Biosciences, Franklin Lakes, NJ	560601
Fc Block Cd16/Cd32	2.4G2	BD Biosciences, Franklin Lakes, NJ	553142
Cd11c	N418	Biolegend, San Diego, CA	117339
Cd64	X54-5/7.1	Biolegend, San Diego, CA	139323

Table S2. Staining panel for hematopoietic stem- and progenitor cells (HSPCs): Long-term hematopoietic stem cells (HSC^{LT}), common myeloid progenitors (CMPs), and terminal myeloid progenitors (TMPs).

Antibody	Clone	Manufacturer	Catalog number
c-kit CD117	2B8	Biolegend, San Diego, CA	105820
Sca-1 Ly6A/E	D7	Biolegend, San Diego, CA	108114
FcγR Cd16/Cd32	2.4G2	BD Biosciences, Franklin Lakes, NJ	553142
LY6C	HK1.4	Biolegend, San Diego, CA	128012
CSF-1R CD115	AFS98	Biolegend, San Diego, CA	135506
Flt3 CD135	A2F10.1	BD Biosciences, Franklin Lakes, NJ	560718
CD34	RAM34	BD Biosciences, Franklin Lakes, NJ	553733

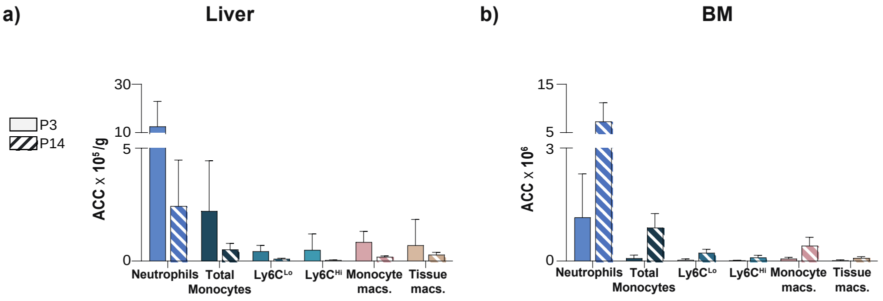

Supplementary Figure S1. Mature myeloid populations contract in the liver and expand in the bone marrow of juvenile mice. Quantification of absolute cell count (ACC) of mature myeloid populations on day 3 (P3 n=3) and 14 (P14 n=3) in the (a) liver and (b) bone marrow (BM). Error bars represent mean ± SD. $Ly6C^{Lo}$ non-classical monocytes ($Ly6C^{Lo}$), $Ly6C^{Hi}$ classical monocytes ($Ly6C^{Hi}$), Macrophages (macs).

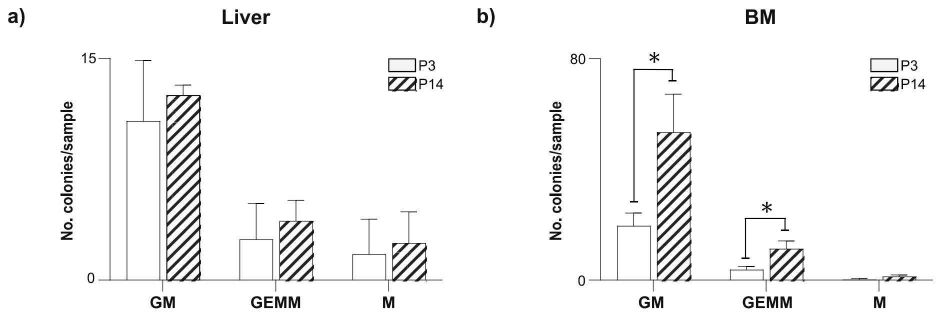

Supplementary Figure S2. HSPCs in the livers of juvenile mice maintain myeloid differentiation capacity. Number (no.) myeloid colony forming units form (a) liver and (b) BM at P3 (liver n=8, BM n=5) and P14 (liver n=2, BM n=3). Granulocyte monocyte, GM; granulocyte, erythrocyte, monocyte, megakaryocyte, GEMM; Megakaryocytes, M. p-value *<0.05. Error bars represent the mean ± SD.
